# Correction: A matter of timing: Biting by malaria-infected *Anopheles* mosquitoes and the use of interventions during the night in rural south-eastern Tanzania

**DOI:** 10.1371/journal.pgph.0006252

**Published:** 2026-04-03

**Authors:** Isaac Haggai Namango, Sarah J. Moore, Carly Marshall, Adam Saddler, David Kaftan, Frank Chelestino Tenywa, Noely Makungwa, Alex J. Limwagu, Salum Mapua, Olukayode G. Odufuwa, Godfrey Ligema, Hassan Ngonyani, Isaya Matanila, Jameel Bharmal, Jason Moore, Marceline Finda, Fredros Okumu, Manuel W. Hetzel, Amanda Ross

The captions for Figs 2-4 are missing from the article. The captions have been provided here:

**Fig 2:**
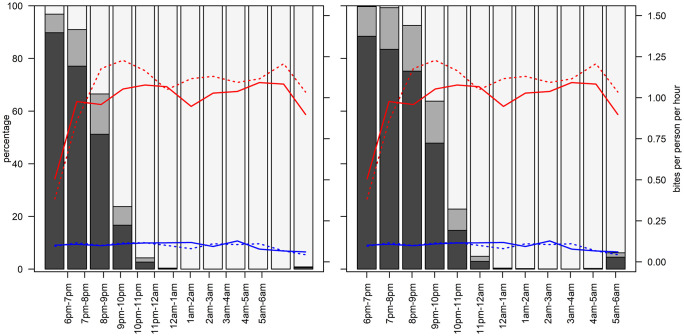
Locations of household members and *Anopheles* bites at night. Left panel: Below school-age, Right panel: school-age and above. Shaded bars: dark grey: outdoors, mid-grey: indoors out of bed, light grey: indoors in bed. Lines: Anopheles bites per hour Blue: An. arabiensis, Red: An. funestus, Solid: indoor HLC, Dotted: outdoor HLC.

**Fig 3:**
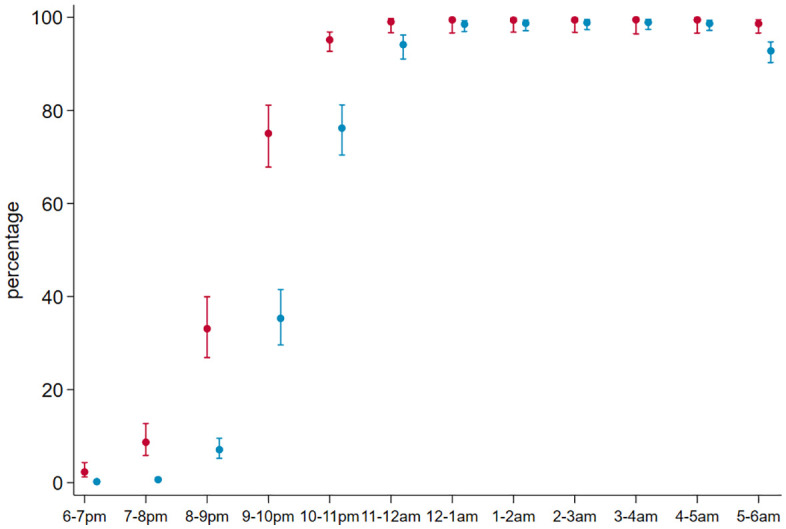
Hourly use of ITNs in the household. Estimated percentage and 95% CI of individuals using ITNs. Red: children below school-age. Blue: school-age and older.

**Fig 4:**
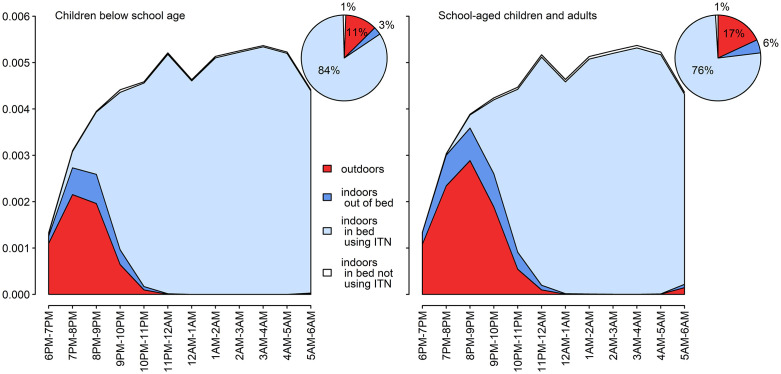
Human exposure to malaria and use of ITNs across the night. The y-axis is the mean number of infected bites per person per hour.
