# Supplementary material for: A matter of timing: Biting by malaria-infected Anopheles mosquitoes and the use of interventions during the night in rural south-eastern Tanzania
Source: PLOS Glob Public Health. 2024 Dec 31;4(12):e0003864. doi: 10.1371/journal.pgph.0003864 (PMC11687804; doi:10.1371/journal.pgph.0003864)
Supplement: S1 Table — (DOCX) [file pgph.0003864.s001.docx]

S1 Table. Proportions of *Anopheles* mosquitoes caught and infected and parous bites early, middle and late at night estimated from different areas.

| \|  \|  \|  \|  \| \| --- \| --- \| --- \| --- \| \|  \| **6-10PM** \| **10PM-2AM** \| **2-6AM** \| \| *Giles, 1957*†* \| \| ***An. gambiae* (Tanga, Tanzania)** \| \| Total catch \| 541 \| 1575 \| 1180 \| \| % of night’s biting \| 16.4 \| 47.8 \| 35.8 \| \| % infected mosquitoes \| 3.6 \| 2.7 \| 1.3 \| \| % of infective bites \| 25 \| 56 \| 19 \| \|  \|  \|  \|  \| \| *Maxwell et al, 1998* \|  \|  \|  \| \| ***An. gambiae* (Muheza, Tanzania)** \| \| Total catch \| 23 \| 189 \| 211 \| \| % of night’s biting \| 5.4 \| 44.6 \| 49.9 \| \| % infected mosquitoes \| 8.7 \| 7.4 \| 4.3 \| \| % of infective bites \| 8 \| 56 \| 36 \| \| ***An. funestus* (Muheza, Tanzania)** \| \| Total catch \| 31 \| 232 \| 86 \| \| % of night’s biting \| 9.8 \| 66.5 \| 24.6 \| \| % infected mosquitoes \| 0 \| 3.0 \| 2.3 \| \| % of infective bites \| 0 \| 78 \| 22 \| \|  \|  \|  \|  \| \| *Bockarie et al, 1996* \|  \|  \|  \| \| ***An. gambiae* (Bayama Sierra Leone)** \|  \|  \|  \| \| Total catch \| 104 \| 2016 \| 4373 \| \| % of night’s biting \| 1.6 \| 31.0 \| 67.3 \| \| Parity rate (%) \| 44.0 \| 58.3 \| 66.9 \| \| % of parous bites \| 1 \| 28 \| 71 \| \| ***An. punctulatus* (Yauatong, Papua New Guinea)** \| \| \| \| \| Total catch \| 202 \| 848 \| 1100 \| \| % of night’s biting \| 9.4 \| 39.4 \| 51.2 \| \| Parity rate (%) \| 39.8 \| 54.8 \| 63.2 \| \| % of parous bites \| 6 \| 38 \| 56 \| \| ***An. punctulatus* (East Sepik, Papua New Guinea)** \| \| \| \| \| Total catch \| 535 \| 1794 \| 1839 \| \| % of night’s biting \| 12.8 \| 43 \| 44.1 \| \| *Plasmodium falciparum* \|  \|  \|  \| \| % infected mosquitoes \| 0.7 \| 1.7 \| 2.3 \| \| % of infective bites \| 5 \| 40 \| 55 \| \| *Plasmodium vivax* \|  \|  \|  \| \| Sporozoite rate (%) \| 1.3 \| 0.9 \| 1.1 \| \| % of infective bites \| 16 \| 37 \| 47 \| \|  \|  \|  \|  \| \| *Robert and Carnevale, 1991* \|  \|  \|  \| \| ***An. gambiae* and *An. funestus* (Burkina Faso)** \|  \|  \|  \| \| Total catch \| 5900 \| 13000 \| 10850 \| \| % of night’s biting \| 19.8 \| 43.7 \| 36.5 \| \| % parous \| 22.8 \| 25.2 \| 24.6 \| \| % parous bites \| 18 \| 45 \| 37 \| \| % infected mosquitoes \| 0.05 \| 0.08 \| 0.19 \| \| % of infective bites \| 9 \| 31 \| 61 \| \|  \| 5 \| 36 \| 59 \| |
| --- | --- | --- | --- | --- | --- | --- | --- | --- | --- | --- | --- | --- | --- | --- | --- | --- | --- | --- | --- | --- | --- | --- | --- | --- | --- | --- | --- | --- | --- | --- | --- | --- | --- | --- | --- | --- | --- | --- | --- | --- | --- | --- | --- | --- | --- | --- | --- | --- | --- | --- | --- | --- | --- | --- | --- | --- | --- | --- | --- | --- | --- | --- | --- | --- | --- | --- | --- | --- | --- | --- | --- | --- | --- | --- | --- | --- | --- | --- | --- | --- | --- | --- | --- | --- | --- | --- | --- | --- | --- | --- | --- | --- | --- | --- | --- | --- | --- | --- | --- | --- | --- | --- | --- | --- | --- | --- | --- | --- | --- | --- | --- | --- | --- | --- | --- | --- | --- | --- | --- | --- | --- | --- | --- | --- | --- | --- | --- | --- | --- | --- | --- | --- | --- | --- | --- | --- | --- | --- | --- | --- | --- | --- | --- | --- | --- | --- | --- | --- | --- | --- | --- | --- | --- | --- | --- | --- | --- | --- | --- | --- | --- | --- | --- | --- | --- | --- | --- | --- | --- | --- | --- | --- | --- | --- | --- | --- | --- | --- | --- | --- | --- | --- | --- | --- | --- | --- | --- | --- | --- | --- | --- | --- |
| * Sporozoite test on only gravid mosquitoes  † Indoor resting populations collected by hand  ‡ Host seeking populations collected by CDC light traps  ** Biting populations collected by the human landing catch |
