# Supplementary material for: A matter of timing: Biting by malaria-infected Anopheles mosquitoes and the use of interventions during the night in rural south-eastern Tanzania
Source: PLOS Glob Public Health. 2024 Dec 31;4(12):e0003864. doi: 10.1371/journal.pgph.0003864 (PMC11687804; doi:10.1371/journal.pgph.0003864)
Supplement: Attachment — Submitted filename: Response to Reviewers.docx [file pgph.0003864.s002.docx]

Reviewers' comments:

1. We do not publish any copyright or trademark symbols that usually accompany proprietary names, eg (R), (C), or TM (e.g. next to drug or reagent names). Please remove all instances of trademark/copyright symbols throughout the text, including ® on page 6.

**Copyright and trademark symbols have been deleted from page 6 and throughout the manuscript text**

2. Figure 1: please (a) provide a direct link to the base layer of the map (i.e., the country or region border shape) and ensure this is also included in the figure legend; and (b) provide a link to the terms of use / license information for the base layer image or shapefile. We cannot publish proprietary or copyrighted maps (e.g. Google Maps, Mapquest) and the terms of use for your map base layer must be compatible with our CC-BY 4.0 license.

Note: if you created the map in a software program like R or ArcGIS, please locate and indicate the source of the basemap shapefile onto which data has been plotted.

If your map was obtained from a copyrighted source please amend the figure so that the base map used is from an openly available source. Alternatively, please provide explicit written permission from the copyright holder granting you the right to publish the material under our CC-BY 4.0 license.

Please note that the following CC BY licenses are compatible with PLOS license: CC BY 4.0, CC BY 2.0 and CC BY 3.0, meanwhile such licenses as CC BY-ND 3.0 and others are not compatible due to additional restrictions.

If you are unsure whether you can use a map or not, please do reach out and we will be able to help you. The following websites are good examples of where you can source open access or public domain maps:

* U.S. Geological Survey (USGS) - All maps are in the public domain. (http://www.usgs.gov)

* PlaniGlobe - All maps are published under a Creative Commons license so please cite “PlaniGlobe, http://www.planiglobe.com, CC BY 2.0” in the image credit after the caption. (http://www.planiglobe.com/?lang=enl)

* Natural Earth - All maps are public domain. (<http://www.naturalearthdata.com/about/terms-of-use/>)

**The map in Figure 1 was** **created in QGIS. The source of the base map shapefile onto which data was plotted is** **<https://diva-gis.org/>. This has now been indicated in the figure legend in the manuscript (p34)**

Reviewer's Responses to Questions

1. Does this manuscript meet PLOS Global Public Health’s publication criteria? Is the manuscript technically sound, and do the data support the conclusions? The manuscript must describe methodologically and ethically rigorous research with conclusions that are appropriately drawn based on the data presented.

Reviewer #1: Yes

Reviewer #2: Yes

2. Has the statistical analysis been performed appropriately and rigorously?

Reviewer #1: Yes

Reviewer #2: Yes

3. Have the authors made all data underlying the findings in their manuscript fully available (please refer to the Data Availability Statement at the start of the manuscript PDF file)?

The PLOS Data policy requires authors to make all data underlying the findings described in their manuscript fully available without restriction, with rare exception. The data should be provided as part of the manuscript or its supporting information, or deposited to a public repository. For example, in addition to summary statistics, the data points behind means, medians and variance measures should be available. If there are restrictions on publicly sharing data—e.g. participant privacy or use of data from a third party—those must be specified.

Reviewer #1: Yes

Reviewer #2: Yes

4. Is the manuscript presented in an intelligible fashion and written in standard English?

Reviewer #1: Yes

Reviewer #2: Yes

5. Review Comments to the Author

Reviewer #1: This study combines entomological and human behavior data to assess malaria transmission risk in rural Tanzania. It found that most infective mosquito bites occur indoors at night when people are using insecticide-treated nets (ITNs). High ITN use potentially prevents 76-85% of infective bites, highlighting the importance of maintaining effective ITN coverage.

Here are the key issues the authors should consider addressing in this manuscript:

1. Consistency in data collection timeframes: The entomological data and human behavior data were collected at different times (2019 vs 2016-2017) and in different villages within the same region. This temporal and spatial mismatch should be more explicitly acknowledged as a limitation.

We have added a couple of sentence to emphasize this limitation more fully. The text on this limitation (Tracked changes version page 23, paragraph 1, line 3) now reads:

‘Another limitation was that the two datasets for the entomological and human behaviour data were collected at different times, and from different villages, even though all were in the Kilombero valley. Villages where entomological surveys were carried out were at higher altitudes (range 311 to 1884m above sea level) than villages where human surveys were done ( 255m to 298m) [78]. The human behaviour data was collected between August 2016 and November 2017, and the entomological data between August 2018 and September 2019. If the human and mosquito behaviour both changed over the altitude range or between the years, then the estimates combining the human and mosquito data would not represent any specific location and time period accurately, and generalization would also be limited.’

2. While I appreciate the difficulty in attaining infected mosquitoes, only 356 infected mosquitoes were found, leading to imprecision in estimates for some hours and locations. The authors aggregated across time periods to address this, but should discuss this limitation more thoroughly.

We have added a sentence to emphasize this limitation more fully. The text on this limitation (Page 22, paragraph 3, line 1) now reads:

‘A disadvantage to characterizing risk during the night is the need for sufficient data to characterize each segment of the night. There were only 356 infected mosquitoes in the study. This led to imprecision for some hours and locations for the estimated proportions of mosquitoes infected, and consequently we aggregated across some time-periods. A larger study size would be beneficial, however since the aggregation focused on time periods when there were relatively fewer mosquitoes biting, it is unlikely to substantially alter the estimated peak period for infective bites during the night.’

3. Assumptions in analysis:

- The authors assume 100% protection from ITNs while in use, which is likely an overestimate. This assumption should be more clearly stated and its implications discussed.

We have added a sentence and the text for this limitation (page 22, paragraph 2, line 1) now reads:

‘We assumed that 100% of bites are prevented while under a net. We recognize that this is untrue and represents an upper bound: it is likely that protection starts off reasonably high but less than 100% and wanes as the net ages. Nets have been found to still offer some protection when there is insecticide resistance [74] or when old and with holes [61]. A modelling study would able to predict the proportion of bites prevented over the lifespan of different types of ITNs, taking human behaviour into account.’

- The analysis assumes biting rates are the same across all age groups, which may not be accurate. This limitation should be acknowledged.

This limitation is acknowledged in the discussion (page 22, second paragraph, line 6):

‘We also assume that the biting rates in the entomological study would be the same for all age-groups in the human behaviour study. There is evidence of different biting rates by host size [75, 76], and that residents carrying out activities other than HLC may affect mosquito landing. This would not affect comparisons within the same age-group but would affect comparisons across age-groups and absolute levels of risk.’

4. Limited time frame: The study only covers 6PM to 6AM, potentially missing daytime or early morning biting. This limitation should be noted.

This limitation is noted (Page 22, paragraph 3, line 7):

‘We also do not capture day biting, which has been reported to contribute to transmission in a study in the Central African Republic [77], or early morning biting after 6am. We also cannot capture exposure for people who were away from the household [33].’

5. Self-reported ITN use: The very high reported ITN use (99%) may be influenced by self-reporting bias or the recent distribution campaign. This potential bias should be discussed.

This limitation is now discussed (Page 23, paragraph 2, line 3):

‘The high ITN use in our study area may have occurred for several reasons. It was self-reported, the human behaviour study occurred within two years of a mass distribution campaign and the communities are within an area where several malaria transmission control studies had been carried out and the community may be adequately sensitised about the benefits of nets. This may not apply to the villages where the entomological surveys were carried out. In addition, the human behaviour survey only captures people who are at home. Those who have gone out may be less likely to use a net.’

6. Generalizability: The study was conducted in an area with recent IRS application and high ITN coverage. The authors should discuss how this might limit generalizability to other settings.

We have added a sentence (p23, paragraph 2, line 1):

The high ITN and IRS use in the study area may limit the generalizability of the study findings to areas with lower ITN and IRS coverage since these interventions may impact mosquito biting locations and timing, and potentially also human behaviour.

7. Statistical methods: The authors used different methods to account for clustering in the entomological and human behavior data. They should justify this choice and possibly conduct sensitivity analyses with alternative methods.

We used two different methods for accounting for clustered data based on assumptions underlying the different sampling, although we acknowledge that different choices would also have been valid.

For the entomological data, we used random effects since there was clustering by two things – household and night. Other methods do not allow multiple sources of clustering. Random effects ‘shrinks in’ outlying values towards the mean, which we considered to be reasonable due to the high variation and fluctuations over time. However for the human behavior survey, we decided to account for clustering by inflating the standard errors, allowing outlying households to be unaffected by the body of the data. This also allowed the point estimates to intuitively match those of a simple analysis. We carried out some sensitivity analyses. In general, different analysis choices for accounting for clustering usually do not make very big differences to the estimates and confidence intervals.

The text reads (p 22, paragraph 1, line 8):

‘We needed to account for the variance structure introduced by the cluster sampling in our study, and different analysis methods may lead to different estimates. We used random effects for household and night for the entomological data because our data was unbalanced and our question focused on comparing the biting rates between the hours. We used robust standard errors for the human behaviour data. We investigated alternative methods as a sensitivity analysis: assuming zero for the proportion of mosquitoes infected in the hours with few mosquitoes caught and using robust variance estimates for the entomological data both lead to slightly higher estimates for the proportion of infective bites between 10PM and 5AM.’

8. Outdoor exposure: While the study focuses on indoor biting, more discussion on the implications of the 11-17% outdoor biting could be valuable, especially in the context of residual transmission.

Outdoor biting is mentioned in the text on Page 24, paragraph 2.

‘While in this area, the majority of infective bites could be prevented by the use of mosquito nets while sleeping, a small proportion of the infective bites occurred outdoors before people retired to bed. Outdoor biting needs to be addressed and could be reduced directly by tools designed for this. There is also some evidence that mosquitoes biting outdoors go indoors at least once during their life and can be impacted by indoor interventions [83, 90].

9. Practical implications: The discussion could be strengthened by more explicitly addressing the practical implications of these findings for malaria control programs.

We have tried to summarize the practical implications in the abstract and conclusions. The implications are that ITN remain very important as a strategy in this area, and that there is also a need for outdoor interventions in the evening. Further data and analysis on the timing of infective bites and human behavior in other settings may be useful for designing effective intervention strategies, and this paper contributes to the evidence that there are different biting rates and proportions of mosquitoes infected at different times during the night.

10. Data visualization: Some of the figures, particularly Figure 2, are quite complex and could potentially be simplified or split into multiple figures for clarity.

We acknowledge figure 2 is a little complex. Our reason for plotting mosquito and human behaviour data on the same time scale was to enhance side-by-side visualization of the hourly exposure of humans to *Anopheles* bites. Although we tried separating them, we found it was then hard to combine the human and mosquito information by eye across graphs.

Addressing these issues would strengthen the manuscript and provide a more comprehensive and nuanced presentation of the study's findings and limitations.

Reviewer #2: In general, the MS addresses a very important topic on time and place of risk of exposures to infectious malaria mosquitoes in the communities. This is important for reinforcing use of interventions in the communities and understanding the gaps in the interventions and when and how we can supplement them primary ones (ITNs). The MS is well written, analysis well captured, and limitations of the study well outlined given that this is a secondary analysis, and a study conducted about 8 years ago. One limitation though with most human behavior observations including this study that is not mentioned is that the hourly capture of spatial presence of individuals indoors and outdoors sometimes can overestimate exposure risk for location (indoor or outdoor). This is because an individual can be counted twice or several times in a single period of observation i.e. in that 30-minute observation in this study, an individual can move between outdoors/indoors several times especially in rural settings where housing structures do not contain all the amenities indoors and the way the communities interact and live in these societies. So, I think this should be included as a limitation in this study as well. Otherwise well done team.

Thank you. The human behavior survey collects information only at each half-hour time-point and not between time-points. People may have been outdoors and indoors during the half-hour, but they will only be counted once and in one location. We have added a sentence to clarify this (p7, first paragraph).

6. PLOS authors have the option to publish the peer review history of their article (what does this mean?). If published, this will include your full peer review and any attached files.

Do you want your identity to be public for this peer review? If you choose “no”, your identity will remain anonymous but your review may still be made public.

For information about this choice, including consent withdrawal, please see our Privacy Policy.

Reviewer #1: Yes: Eric Ochomo

Reviewer #2: No
